# Portable Chest Radiograph: A Boon for Critically Ill Patients With COVID-19 Pneumonia

**DOI:** 10.7759/cureus.36330

**Published:** 2023-03-18

**Authors:** Yash Jakhotia, Avinash Dhok, Priyanka Mane, Kajal Mitra

**Affiliations:** 1 Radiodiagnosis, NKP Salve Institute of Medical Sciences and Research Centre (IMSRC) and Lata Mangeshkar Hospital, Nagpur, IND

**Keywords:** mortality, air space opacities, critically ill patients, portable chest radiograph, covid-19 pneumonia

## Abstract

Objective: In the present study, we evaluated the role of portable chest radiographs in critically ill patients with COVID-19 pneumonia in whom computed tomography (CT) of the chest was not feasible.

Methods: A retrospective chest X-ray study of patients under investigation for COVID-19 was performed in our dedicated COVID hospital (DCH) during the exponential growth phase of the COVID-19 outbreak (August-October, 2020). A total of 562 on-bed chest radiographs were examined comprising 289 patients (critically ill who couldn’t be mobilized for CT) along with positive reverse transcription-polymerase chain reaction (RT-PCR) tests. We categorized each chest radiograph as progressive, with changes, or improvement in appearance for COVID-19, utilizing well-documented COVID-19 imaging patterns.

Results: In our study, portable radiographs provided optimum image quality for diagnosing pneumonia, in critically ill patients. Although less informative than CT, nevertheless radiographs detected serious complications like pneumothorax or lung cavitation and estimated the evolution of pneumonia.

Conclusion: A portable chest X-ray is a simple but reliable alternative for critically ill SARS-CoV-2 patients who could not undergo chest CT. With the help of portable chest radiographs, we could monitor the severity of the disease as well as different complications with minimal radiation exposure which would help in identifying the prognosis of the patient and thus help in medical management.

## Introduction

After first appearing in Wuhan, China at the end of 2019, the severe acute respiratory syndrome coronavirus 2 (SARS-CoV-2)-caused coronavirus disease 2019 (COVID-19) has continued to spread unchecked throughout the world. The World Health Organization (WHO) situation report states that as of October 23, 2022, more than 630 million persons had been proven positive globally [1]. The Coronaviridae family includes SARS-CoV-2 [2,3]. COVID-19-positive patients show a wide range of clinical spectrum ranging from asymptomatic carriers to patients requiring intensive care [4,5].

Ground glass opacities with a peripheral and basal predominance form major imaging findings of COVID-19 as the initial manifestation of the disease. These imaging findings closely resemble other viral pneumonia. As the disease progresses these ground glass opacities transform into consolidatory changes [6].

Chest computed tomography (CT) is widely used for the assessment of the severity of COVID-19 infection. It helps clinicians widely in classifying patients into different categories according to the severity of disease which further aids them in management protocols. Different complications can be identified on CT scans which could complicate the disease course even in the early phase [7,8]. However, some patients present as emergencies, in which case performing CT becomes necessary but challenging because the patient cannot be removed from the intensive care unit. Mobile X-rays are an alternative in such cases.

A wide range of radiological findings is seen on chest X-rays in the case of COVID-19-positive patients, including ground glass opacities, consolidatory changes, and linear opacities. Sometimes pleural effusion, nodular lung lesions, and even pneumothorax can be seen. These findings are usually seen involving the bilateral lung parenchyma predominantly involving peripheral and basal zones, however, involvement could also be seen involving unilateral lung parenchyma [9,10].

The present study aims to determine the utility of portable chest X-rays in critically ill patients with assisted ventilation.

## Materials and methods

Data collection

The study was conducted after obtaining written permission from our institutional ethics committee. From August 1 to October 31, 2020, chest X-rays of 289 symptomatic critically ill SARS-CoV-2 patients with ICU admission were gathered and analyzed using the picture archiving and communication system (PACS).

Study design

Retrospective study.

Study setting

Department of Radiodiagnosis at NKP Salve Institute of Medical Sciences and Research Centre and Lata Mangeshkar Hospital, Nagpur, India.

Study period

Three months (August 2020 to October 2020)

Study population

All patients positive for COVID-19 pneumonia by reverse transcription-polymerase chain reaction (RT-PCR) or rapid antigen test and require assisted ventilation and advised portable chest X-rays during the study period are included in the study. Follow-up X-rays were advised to patients whenever clinically indicated and the findings were compared to previous X-rays.

Imaging instrument and technique

Radiographs were taken using, "Allengers MARS 15/30 High-Frequency Mobile X-Ray Machine, 300mA". All the radiographs were taken in AP view with the patient in a supine position with exposure factors of around 100-110 kVp and 4-8 mAs. A detector size of 43 x 35 cm was used. The centering point was around the 7th thoracic vertebra.

Inspection

The chest X-rays were evaluated by a trained radiologist having experience of a minimum of three years in thoracic imaging. Imaging findings were interpreted and further patients were classified into different categories as per severity. In follow-up X-rays, findings were classified as progression, regression of the disease, or no change. Imaging findings were majorly classified as ground glass opacities, consolidation, pleural effusion, atelectasis, and reticular opacities.

Ethics approval

The study was approved by the Medical Ethics Committee of NKP Salve Institute of Medical Sciences and Research Centre with the letter number: (NKPSIMS & RC and LMH/IEC/36/2020).

## Results

This study analyzed 289 medical X-rays taken of patients aged 35-85 years old with a mean age of 58 years. The ratio of male to female population was approximately similar with 150 male patients and 139 female patients (Table 1).

**Table 1:** Demographics of 289 patients with COVID-19 pneumonia using portable chest radiographs.

Demographic criteria	Total number of patients (N=289)
Mean age (years)	58
Age group (years)	35-85
Male: Female	150:139

Of the 289 patients, 132 succumbed to death (approximately 45%), and 157 survived (Table 2). All patients were critically ill, and different types of ventilatory methods were used for treatment purposes: invasive ventilation (50%), non-invasive ventilation (30%), and high-flow oxygen inhalation (20%) (Table 3).

**Table 2:** Outcome of 289 patients with COVID-19 pneumonia.

Outcome	Total number of patients (N=289)
Non-survivors	132 (45%)
Survivors	157 (55%)

**Table 3:** Types of ventilatory supports used among survivors.

Types of ventilation	Total number of patients (N=289)
Invasive ventilation	144 (50%)
Non-invasive ventilation	87 (30%)
High-flow oxygen inhalation	58 (20%)

Out of a total of 289 patients, follow-up X-rays were performed on a total of 129 patients (45), while the remaining 160 patients (55%) underwent a single X-ray (Table 4).

**Table 4:** Number of portable chest radiographs performed per patient.

No. of X-rays per patient	Total number of patients (N=289)
1	160 (55%)
2	68 (24%)
3	33 (11%)
≥4	28 (10%)

The majority of the X-rays showed extensive ground glass opacities, while other findings included consolidatory changes (62%), pleural effusion (18%), atelectasis (4%), and reticular opacities (2%). Ground glass opacities were the most common finding seen followed by consolidatory changes (Table 5).

**Table 5:** Findings observed in portable chest radiographs.

Parameters	Total number of patients (N=289)
Ground glass opacities	289 (100%)
Consolidation	180 (62%)
Pleural effusion	53 (18%)
Atelectasis	14 (4%)
Reticular opacities	6 (2%)

Of the 273 follow-up X-rays, 131 (47%) showed a significant increase compared to the prior X-rays, while 58 (21%) showed no changes and 84 (30%) showed improvement (Table 6). Among non-survivors, 62 patients died without follow-up X-rays within the first four days of admission. Among the survivors (n = 157), follow-up X-rays were done in 111 patients with 198 radiographs. Out of these, 66 (33%) showed an increase in the severity of pneumonia.

**Table 6:** Changes in follow-up chest radiographs.

Findings compared to the previous X-ray	Number (N=273)
Deterioration	131 (47%)
No change	58 (21%)
Improvement	84 (30%)

This study provides insight into how X-rays can provide an accurate picture of the severity of pneumonia and can help guide treatment decisions. It also clearly shows the importance of timely follow-up X-rays to monitor the progress of the disease and ensure appropriate treatment is given.

## Discussion

Although X-rays are less accurate than CT scans, they can nevertheless identify significant complications like pneumothorax or cavitary lesions and predict the course of pneumonia. In severely ill patients, it is thus a straightforward effective alternative to determine the severity of the disease and its complications in patients who were unable to have a chest CT [11].

The presence of bilateral ground-glass opacities or consolidatory changes on a chest radiograph in an appropriate clinical setting is highly suggestive of COVID-19 infection [12]. To make a diagnosis, it should be utilized in combination with a clinical examination, especially when it is difficult to move such critically ill patients and occasionally when rapid and reliable serologic testing is not available.

The major drawback of X-ray imaging remains the soft tissue of the chest wall (obesity, breast tissue), preventing the X-ray beam from penetrating the body in the supine position. This imitates peripheral, basilar airspace opacity which manifests as false positive results. Furthermore, numerous pathologic conditions exhibit the same disease pattern that we have identified as being specific to COVID-19 [13]. Its close differentials include diseases such as eosinophilic pneumonia, non-specific interstitial pneumonia, and conditions causing acute lung injury. It can become difficult to differentiate these conditions from COVID-19 pneumonia. As a result, we don't recommend that this pattern's peculiarity should always imply COVID-19 positivity [14].

**Figure 1:**
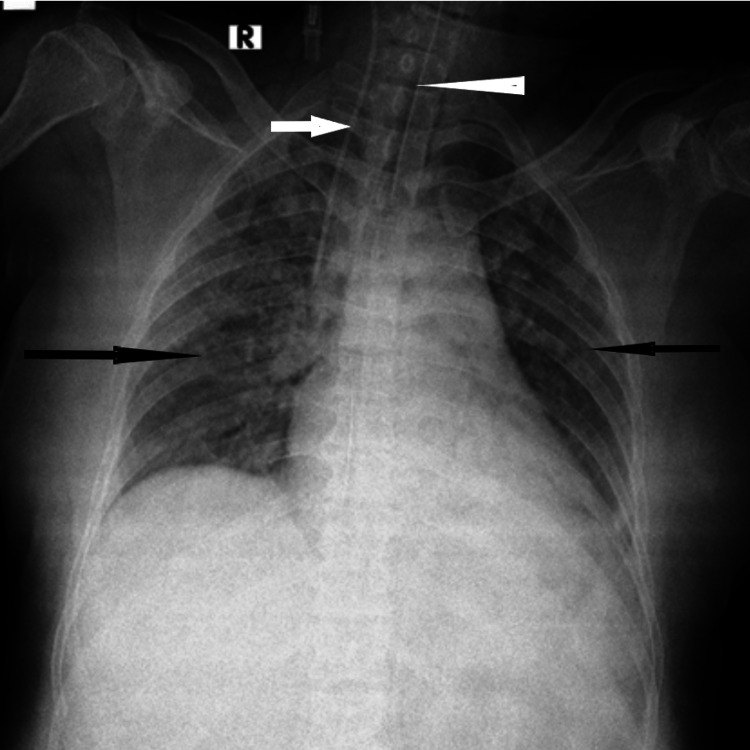
Case 1 Thirty-five-year-old COVID-19-positive patient had a history of dyspnoea for 10 days. The patient underwent invasive ventilation. Diffuse patchy air space opacities indicated by black arrows are noted involving bilateral lung parenchyma on portal chest X-ray suggestive of pneumonia. The central line indicated by a white arrow and the endotracheal tube indicated by an arrowhead can be seen.

**Figure 2:**
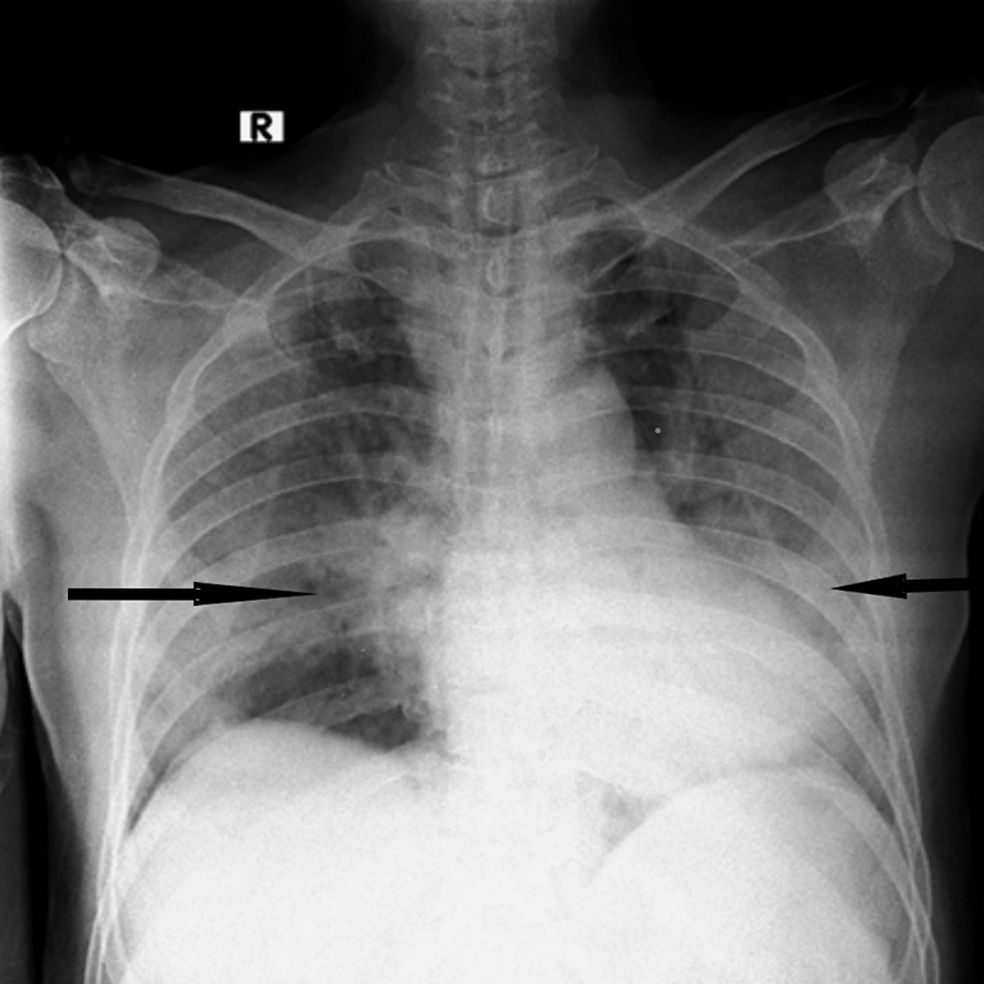
Case 2 Fifty years old COVID-19-positive patient came with complaints of fever, cough, and breathlessness. Her oxygen saturation was 70% under room air, thus high-flow oxygen inhalation was used. She underwent a portable X-ray, which showed consolidatory changes involving bilateral middle lung zones and left lower zones indicated by black arrows.

**Figure 3:**
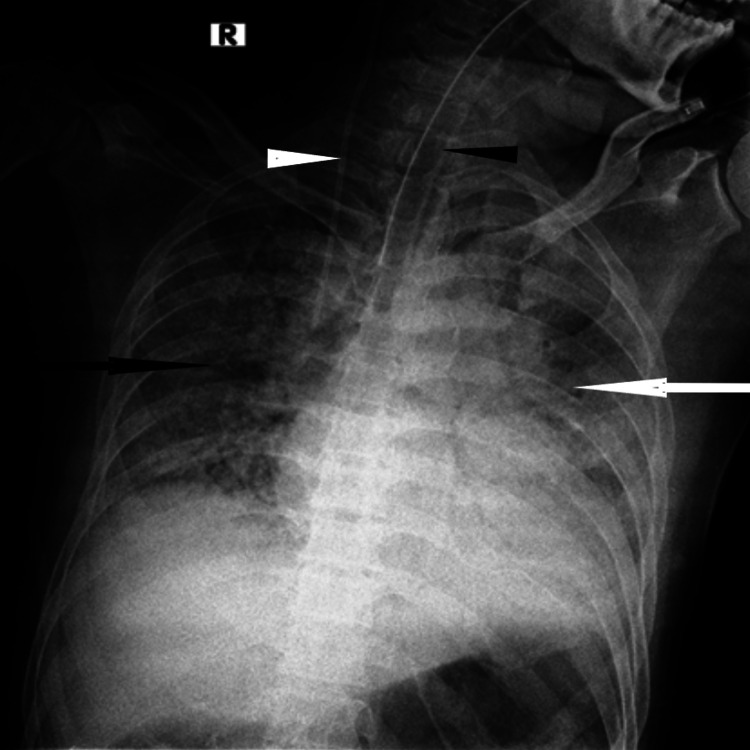
Case 3 Seventy-five years old COVID-19-positive patient had a history of fever and dyspnoea for 10 days. The patient underwent invasive ventilation. The white arrow shows diffuse consolidatory changes involving left lung parenchyma. The black arrow points to ground glass opacities involving the right lung parenchyma. Chest X-ray findings were suggestive of extensive pneumonia. The central line pointed by the white arrowhead and the endotracheal tube pointed by the black arrowhead can be seen.

We further discuss the importance of portable chest radiographs with detailed history and examples highlighted in Figures 1-3.

**Figure 4:**
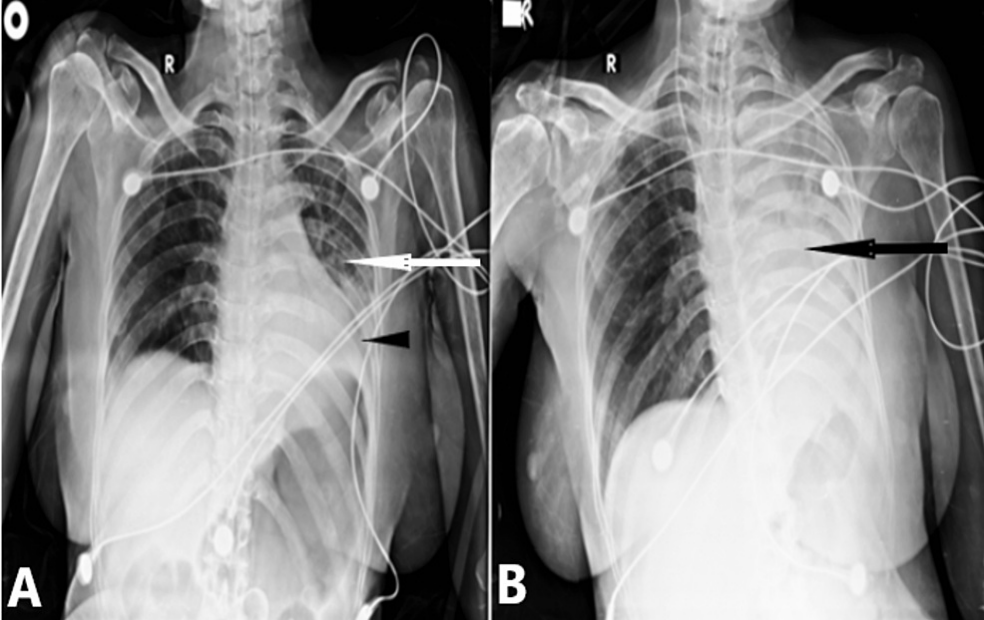
Case 4 A 45-year-old COVID-19-positive patient came with complaints of fever and cough for six days with on-admission oxygen saturation of 60% on room air. She underwent invasive ventilation. (A) On admission, a chest radiograph shows airspace opacities in the left upper and middle lung zones pointed by a white arrow suggestive of patchy consolidatory changes with mild left-sided pleural effusion shown by a black arrowhead. (B) Follow-up X-ray after five days showed a white-out left lung parenchyma indicated by a black arrow suggestive of massive pleural effusion. The patient succumbed to death three days later because of respiratory failure.

**Figure 5:**
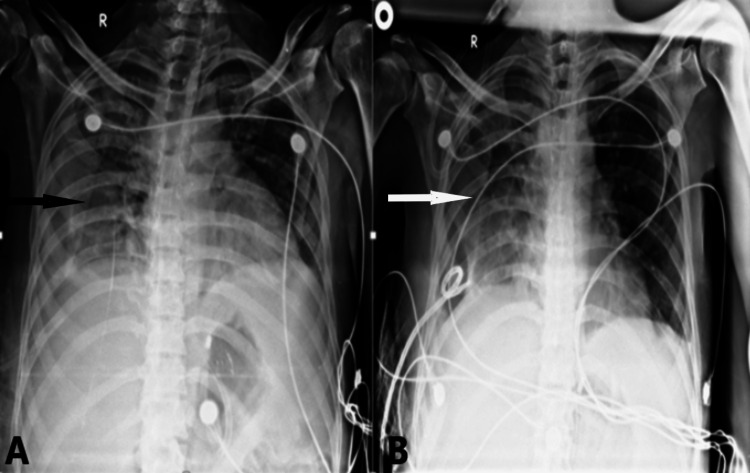
Case 5 A 50-year-old COVID-19 positive came with complaints of fever and breathlessness for three days. SpO2 on admission was 75%. (A) On admission chest radiograph, black arrow shows diffuse airspace opacities involving right lung parenchyma suggestive of consolidatory changes. (B) Follow-up X-ray after two days showed a resolution of consolidatory changes indicated by the white arrow. The patient was discharged five days later.

Correlation with clinical and imaging findings becomes important to arrive at a diagnosis. At times when it is not possible and reasonable to perform a CT chest, a chest X-ray proves to be a promising tool. Serial follow-up X-rays help in identifying the course of the disease and thus help in clinical management. It also prevents repeated chest CT and radiation exposure too. Examples of the importance of follow-up X-rays are illustrated by the examples given ahead in Figures 4-5.

This study has addressed the importance of portable chest radiographs in ICU setups where the patient cannot be mobilized in improving the prognosis as well as early detection of complications. Finally, as more knowledge about SARS-CoV-2 is gained, the disease's pathogenesis is better understood, and RT-PCR tests continue to advance, chest radiography results may be correlated.

One of the drawbacks of our study remains the poor quality of X-rays obtained because of the patient's position and difficulty in penetration because of the soft tissue of the chest wall. This drawback was minimized by taking X-rays with appropriate exposure factors.

With help of this study, we evaluated both importance of portable chest X-rays as well as the value of follow-up X-rays given patients' clinical symptoms.

## Conclusions

This study helps in finding the out importance of portable chest radiographs in COVID-19-positive patients in critically setting where CT scans cannot be done. By utilizing portable chest radiographs, medical professionals can determine the severity of a disease and track its progression. Additionally, this diagnostic tool can detect potentially fatal complications, providing valuable information to both doctors and patients.

## References

[1] (2022). WHO Coronavirus (COVID-19) Dashboard. https://covid19.who.int.

[2] Chen Y, Li L (2020). SARS-CoV-2: virus dynamics and host response. Lancet Infect Dis.

[3] Huang C, Wang Y, Li X (2020). Clinical features of patients infected with 2019 novel coronavirus in Wuhan, China. Lancet.

[4] Kong WH, Li Y, Peng MW, Kong DG, Yang XB, Wang L, Liu MQ (2020). SARS-CoV-2 detection in patients with influenza-like illness. Nat Microbiol.

[5] Yang Y, Yang M, Yuan J (2020). Laboratory diagnosis and monitoring the viral shedding of SARS-CoV-2 infection. Innovation (Camb).

[6] Parry AH, Wani AH (2020). Pulmonary embolism in coronavirus disease-19 (COVID-19) and use of compression ultrasonography in its optimal management. Thromb Res.

[7] Chung M, Bernheim A, Mei X (2020). CT imaging features of 2019 novel coronavirus (2019-nCoV). Radiology.

[8] Kwee TC, Kwee RM (2020). Chest CT in COVID-19: what the radiologist needs to know. Radiographics.

[9] Wu G, Li X (2020). Mobile X-rays are highly valuable for critically ill COVID patients. Eur Radiol.

[10] Cleverley J, Piper J, Jones MM (2020). The role of chest radiography in confirming covid-19 pneumonia. BMJ.

[11] de Barry O, Obadia I, El Hajjam M, Carlier RY (2020). Chest-X-ray is a mainstay for follow-up in critically ill patients with covid-19 induced pneumonia. Eur J Radiol.

[12] Rousan LA, Elobeid E, Karrar M, Khader Y (2020). Chest x-ray findings and temporal lung changes in patients with COVID-19 pneumonia. BMC Pulm Med.

[13] Cozzi D, Albanesi M, Cavigli E (2020). Chest X-ray in new Coronavirus Disease 2019 (COVID-19) infection: findings and correlation with clinical outcome. Radiol Med.

[14] Livrieri F, Ghidoni G, Piro R (2020). May 2020: is it always COVID-19 no matter what?. Int Med Case Rep J.

